# Validation of the Combined Model Based on Platelet Count and Albumin to Rule out High-Risk Varices in Liver Cirrhosis

**DOI:** 10.1155/2020/5783748

**Published:** 2020-07-13

**Authors:** Zhihui Duan, Li Li, Jinlong Li, Shengyun Zhou

**Affiliations:** ^1^Department of Endoscopy, Xingtai People's Hospital, Xingtai, 054000 Hebei Province, China; ^2^Department of Gastroenterology, Beijing Shijitan Hospital, Capital Medical University, Beijing 100038, China; ^3^Clinical Laboratory, Xingtai People's Hospital, Xingtai, 054000 Hebei Province, China

## Abstract

**Background:**

The Baveno VI criteria based on platelet count and liver stiffness, measured by transient elastography (TE), have been proposed to rule out high-risk varices (HRV) defined as medium or large-sized varices or the presence of high-risk stigmata (cherry red spots and red wale marks). However, TE is not available in all hospitals. Recently, the Rete Sicilia Selezione Terapia hepatitis C virus (RESIST-HCV) criteria recommended that cirrhotic patients with a platelet count > 120000/*μ*L and serum albumin > 36 g/L could avoid esophagogastroduodenoscopy (EGD) screening for HRV.

**Aim:**

We aimed to validate the performance of the RESIST-HCV criteria in two cohorts predominantly characterized with hepatitis B infection.

**Methods:**

Patients with compensated cirrhosis who had blood tests within three months of performing EGD and TE were enrolled retrospectively from two centers. RESIST-HCV criteria were applied to identify patients who did not require EGD screening.

**Results:**

This study included 188 patients from the Xingtai cohort (28 (14.9%) with HRV) and 104 patients from the Beijing cohort (19 (18.3%) with HRV). Of the patients who met the RESIST-HCV criteria (83 in the Xingtai cohort and 26 in the Beijing cohort), 0 and 1 had HRV, respectively, accounting for 44.1% (Xingtai cohort) and 25% (Beijing cohort) of endoscopies that were unnecessary. In the combined cohort, 109 (37.3%) patients met the RESIST-HCV criteria, only 1 (0.9%) HRV was missed, and the negative predictive value was 99.1%. Baveno VI and Expanded Baveno VI criteria spared 15.6% and 23.3% of EGDs, respectively, while missing 0% and 4.8% of HRV, respectively.

**Conclusions:**

In our population, the combined criteria based on platelet count and serum albumin performed well, saving 30-40% of EGDs and correctly identifying 99.1% of patients who could safely avoid screening endoscopies for high-risk varices in compensated cirrhotic patients.

## 1. Introduction

Portal hypertension (PH) is a common complication of liver cirrhosis, and it promotes the transition from the preclinical to the clinical phase of liver cirrhosis. Gastroesophageal varices (GEV) are a major and feared complication of PH, occurring in up to 60% of patients with cirrhosis [1]. Bleeding from GEV occurs as a severe and life-threatening complication of PH [2], with an extremely high risk of death. In particular, bleeding from GEV still has a mortality rate of 10%-15%, despite the clinical progress [3]. Prevention and treatment of variceal bleeding largely depends on the timely diagnosis and risk stratification of GEV [4, 5]. Esophagogastroduodenoscopy (EGD) remains the gold standard diagnostic method for GEV and should be performed to screen for the presence of GEV in all patients who are first diagnosed with liver cirrhosis, in accordance with the recent Baveno VI consensus [5]. However, a variable proportion of cirrhotic patients will not have GEV, as 30%-40% of all varices and 6%-20% of HRV are seen in compensated cirrhosis [3, 6]. Thus, screening all cirrhotic patients with EGD leads to a large number of unnecessary endoscopies, which increases the healthcare costs and the financial burden to the families and societies [7], and has a severe influence on the quality of life of patients. In addition, EGD is invasive, expensive, poorly accepted by patients, and unavailable in developing countries and rural areas [8]. Consequently, there have recently been significant updates in the noninvasive prediction of GEV, especially the use of noninvasive tests (NITs) to assess the likelihood of GEV and HRV [9]. NITs such as assessment of platelet count, spleen diameter, and liver stiffness can help identify patients at very low risk of having HRV or GEV [9–12]. Among them, the Baveno VI criteria (liver stiffness measurement (LSM) < 20 kPa and platelet count > 150000/*μ*L) are the most widely studied and employed, and these criteria are associated with<5% chance of missing HRV and can spare about 30% of EGD in compensated patients [5]. However, as transient elastography (TE) is not widely available in all liver units, the Baveno VI criteria cannot be applied in many clinical settings. The development of noninvasive criteria that do not include TE is desirable. Therefore, an easy-to-use Rete Sicilia Selezione Terapia hepatitis C virus (RESIST-HCV) criteria, which uses only platelet count and serum albumin, have been proposed to exclude HRV in compensated cirrhosis by Calvaruso et al. [13]. By using these criteria, the spared EGD rate and the missed HRV rate were 31.4% and 1.6%, respectively [13].

The primary aim of this study was to validate the performance and safety of the RESIST-HCV criteria compared to screening endoscopy for HRV. The secondary aim was to assess the performance of the Baveno VI and Expanded Baveno VI criteria.

## 2. Materials and Methods

### 2.1. Study Population

This was a retrospective study involving all patients with compensated cirrhosis who underwent EGD from January 2018 to January 2020 and who were referred to Xingtai People's Hospital or Beijing Shijitan Hospital. The data collected included LSM (measured by TE), laboratory tests, liver ultrasonography findings, liver function tests, platelet counts, and EGD results.

### 2.2. Ethics

This study was conducted in compliance with the Declaration of Helsinki and approved by the Ethics Committees at Xingtai People's Hospital and Beijing Shijitan Hospital. Given the retrospective nature of this study, obtaining informed consent was not applicable.

### 2.3. Inclusion Criteria

Patients with Child-Pugh A and B cirrhosis with NITs (laboratory tests, reliable LSM, and ultrasonography) performed within 3 months of EGD were included in the study. A diagnosis of cirrhosis was established based on the history of chronic liver disease, clinical manifestations (especially PH-related complications), liver and spleen ultrasonography and computed tomography findings, presence of GEV on EGD, LSM > 10 kPa, and previous liver biopsy if available.

### 2.4. Exclusion Criteria

Exclusion criteria were the occurrence of decompensation events (ascites, hepatic encephalopathy, Child-Pugh C, previous variceal bleeding, esophageal varices (EV) band ligation, portal vein thrombosis, transjugular intrahepatic portosystemic shunt, and hepatocellular carcinoma), current use of nonselective beta-blockers and antiplatelet agents, anticoagulation, and incomplete data.

### 2.5. Liver Stiffness Measurements

TE was only available for the Beijing cohort. LSM was assessed according to the manufacturer's FibroScan standard procedure [14, 15], performed by one expert operator (Li Li) at Beijing Shijitan Hospital (>100 procedures). LSM was considered valid when there were at least 10 measurements with an interquartile range to median ratio (IQR/M) ≤ 30% [16]. Patients fasted for four hours before the procedure.

### 2.6. Upper Gastrointestinal Endoscopy

Two experienced endoscopists reviewed all the endoscopic findings and assessed the presence and size of GEV independently, without knowledge of the TE and blood test results and clinical data. The presence and size of EV were assessed according to the proposed guidelines [5]. Gastroesophageal varices were defined as low-risk varices (LRV) or high-risk varices (HRV). HRV were defined by a medium or large size or the presence of high-risk stigmata (cherry red spots and red wale marks) [5].

### 2.7. Laboratory Markers

Blood samples were drawn in the fasting state and handled according to the standard procedures of each hospital. The index blood samples chosen for assessing the proposed criteria were the closest to the screening endoscopy (within 3 months).

### 2.8. Statistical Analysis

Continuous data were all expressed as median with interquartile range (IQR), as none were normally distributed. Categorical data were expressed as numbers and percentages. A two-tailed *P* value of less than 0.05 was considered statistically significant. LSM and laboratory data were compared between patients with and without HRV; continuous data were compared using Mann–Whitney *U* test, and Fisher's exact test was used for proportions for categorical data. The rate of spared EGD was calculated as the ratio of the numbers of patients with EGD that could be spared to the total number of patients. The missed HRV rate was defined by the rate of patients with missed HRV among the patients with spared EGD. Sensitivity, specificity, positive predictive value (PPV), and negative predictive value (NPV) were calculated. Statistical analysis was performed with the Statistical Package for Social Sciences (SPSS) version 20.0 (SPSS, Chicago, IL).

## 3. Results

### 3.1. Study Population

**Figure 1:**
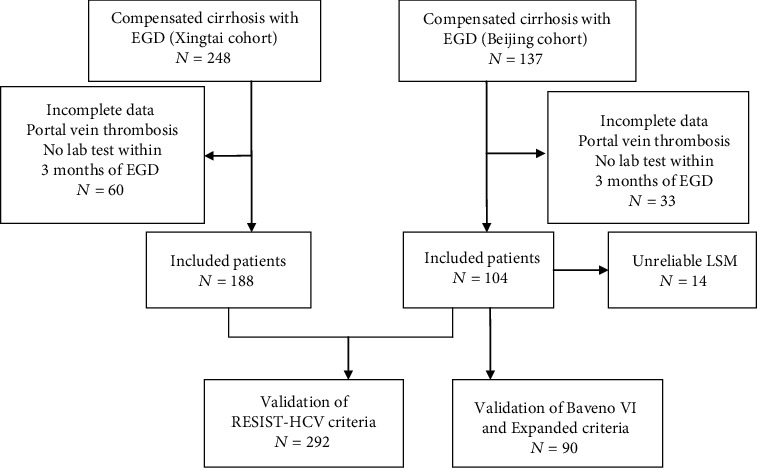
Flow chart of patients included in this study. Abbreviations: EGD: esophagogastroduodenoscopy; LSM: liver stiffness measurement.

Over the study period, 137 compensated cirrhotic patients in the Beijing cohort and 248 patients from the Xingtai cohort underwent EGD. After excluding incomplete data, portal vein thrombosis, and unavailable lab tests within 3 months of EGD, a total of 292 patients from the two cohorts were included to validate the RESIST-HCV criteria, and 90 patients from the Beijing cohort were included to validate the Baveno VI and Expanded Baveno VI criteria. The flowchart of this study is shown in Figure 1. The baseline characteristics of the 292 patients with compensated cirrhosis are shown in Table 1. Overall, HRV was present in 16.1% (47 of 292 cases). The etiology of the underlying liver disease was hepatitis B virus (HBV) in 174 (59.6%), alcohol-related liver disease (ALD) in 12 (4.1%), primary biliary cholangitis (PBC) in 8 (2.7%), hepatitis C virus (HCV) in 4 (1.4%), and 93 others (31.8%); these others included unknown causes in 52, Budd-Chiari syndrome in 14, autoimmune hepatitis in 11, nonalcoholic fatty liver disease (NAFLD) in 9, drug reaction in 6, and overlap syndrome in 1. The majority of patients were Child-Pugh A (245; 83.9%), with 47 cases (16.1%) who were Child-Pugh B. In total, 90 patients had a reliable LSM from Beijing Shijitan Hospital. There were 109 (37.3%) patients who fulfilled the RESIST-HCV criteria ruling out the presence of HRV and could have avoided screening endoscopy. Only one patient with HRV was missed. The missed HRV rate was 0.9% and the NPV was 99.1%. The above data is summarized in Table 1. When compared to patients without HRV, those with HRV had lower platelet count (77 × 10^9^/L (57.5 − 96 × 10^9^/L) vs. 119 × 10^9^/L (90 − 169 × 10^9^/L); *P* < 0.001) and lower serum albumin (37.2 g/L (33.2-40.9 g/L) vs. 41.6 g/L (36.8-44.4 g/L); *P* < 0.001), as seen in Table 2.

### 3.2. Diagnostic Accuracy of RESIST-HCV Criteria for HRV

**Figure 2:**
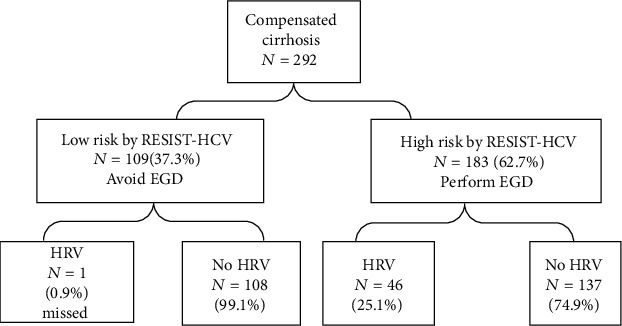
Application of RESIST-HCV criteria to a real-world cohort with compensated cirrhosis. Abbreviations: EGD: esophagogastroduodenoscopy; HRV: high-risk varices.

The RESIST-HCV criteria combine platelet count > 120000/*μ*L and albumin > 36 g/L. In the combined cohort, 109 (37.3%) cases met these criteria, of whom 1 (0.9%) had HRV. Among the 183 (62.7%) cases that did not meet these criteria, 46 (25.1%) had HRV (Figure 2). The combination of platelet count and albumin using the recommended cut-off values to predict HRV gave a sensitivity of 97.9%, specificity of 44.1%, PPV of 25.1%, and NPV of 99.1% (Table 3). One case (0.9%) of HRV was missed (Figure 2), and the case had liver cirrhosis secondary to HBV, and the platelet count and albumin were 142000/*μ*L and 40.5 g/L, respectively.

### 3.3. Analysis of the Avoidance of the Baveno VI, Expanded BavenoVI Criteria, and RESIST-HCV Criteria

Using the RESIST-HCV criteria, we classified all patients into low risk (those who fulfilled these criteria) and high risk (those who did not fulfill these criteria). The RESIST-HCV criteria could spare 37.3% (109 of 292) of EGDs, with a 0.9% (1 of 109) missed HRV rate and NPV of 99.1% (Table 3). Of the 90 patients who had reliable LSM from the Beijing cohort, 14 (15.6%) and 21 (23.3%) patients met the Baveno VI and the Expanded BavenoVI criteria, respectively, and 0% (0 of 14) and 4.8% (1 of 21) of HRV were missed, respectively. The RESIST-HCV criteria had the best performance with an area under receiving operator characteristics curve (AUROC) of 0.710 (Table 3).

## 4. Discussion

In the present study, we validated the recently published RESIST-HCV criteria [13] that use only platelet count and serum albumin level to identify patients who are at low risk of HRV and can safely avoid endoscopic screening, saving time and reducing costs. This is the first validation performed in Chinese patients. Interestingly, the main etiology of cirrhosis was HBV, which makes this study different from a previous study [13], where the main etiology was HCV. Compared with the study with HCV predominance, our study demonstrated that these criteria had a similar diagnostic accuracy for HBV-related cirrhosis patients. Applying these criteria in our study would have spared 37.3% of endoscopies, with a 0.9% missed HRV rate.

As expected, in our study, the RESIST-HCV criteria could safely avoid 37.3% (109 of 292) of EGDs, while maintaining the missed HRV rate below 5%, which was similar to the recent study by Calvaruso et al. [13]. In the large cohort of 1381 cirrhotic patients with HCV, the RESIST-HCV criteria spared 31.4% of EGDs and showed a 1.6% false-negative rate for the medium and large varices [13]. Similarly, the RESIST-HCV criteria failed to identify one (0.9%) HBV patient with HRV. To our knowledge, the RESIST-HCV criteria are clearly able and safe to stratify compensated cirrhotic patients for HRV risk. In the present study, the RESIST-HCV criteria were the most accurate diagnostic tool for ruling out HRV patients.

We further validated the Baveno VI and the Expanded Baveno VI criteria. In this study, the Baveno VI criteria were safe and 15.6% of patients could have avoided endoscopy, while the risk of missing HRV was 0%, and the NPV was 100%. The spared EGD rate (15.6%) was comparable with that reported in previous studies [7, 13, 17–20]. In addition, our data demonstrated an acceptable rate of missing HRV. The Expanded Baveno VI criteria would have spared 23.3% of unnecessary EGDs and missed 4.8% of HRV. The number of spared EGDs is lower than the number reported in previous studies [7, 13, 14, 19]. The lower number of spared EGDs may be explained by the bias in selection of patients and the high prevalence of HRV, which may have led to a low NPV and influenced the diagnostic performance [18]. Besides, the sample size (*n* = 90) is rather small. In addition, identifying and classifying varices in cirrhosis by different endoscopists may be inconsistent due to differences in technique and the experience of doctors [21].

Our study had a few limitations. First, the study was retrospective and the EV size and high-risk stigmata were evaluated by two experienced endoscopists. However, this issue was present in other studies [11, 13, 18–20, 22]. Most studies that attempted to noninvasively rule out HRV, however, were retrospective and did not include an assessment of EV size [23]. Reassuringly, the diagnostic performance of the RESIST-HCV criteria was consistent with the performance reported in the recently published study [13]. Second, LSM measured by TE is useful for the assessment of HRV. However, TE was only available for 90 patients from Beijing Shijitan Hospital. Third, the present study lacked internal and external validation sets. Further validation in larger cohorts is needed.

## 5. Conclusions

Our study validated the RESIST-HCV criteria which could identify low-risk patients who can safely circumvent surveillance endoscopy for HRV screening for more than 30% of EGDs by using simple-to-use laboratory parameters not requiring TE. However, we have to acknowledge that a small proportion of HRV cases will be missed with an acceptable rate. Prospective validation of these criteria would be required to prove its diagnostic performance for HRV in other populations and various etiologies.

## Figures and Tables

**Table 1:** Main characteristics of the study.

Variables	Total cohort, *n* = 292	Beijing cohort, *N* = 104	Xingtai cohort, *N* = 188
Male (%)	187 (64.0)	57 (54.8)	130 (69.1)
Age (years)	52 (43-60.5)	52 (43-60)	52.5 (43-61.5)
BMI	23.2 (21.4-25.2)	23.2 (21.4-25.2)	NA
Etiology			
Hepatitis B	174 (59.6)	36 (34.6)	138 (73.4)
Hepatitis C	4 (1.4)	0 (0)	4 (2.1)
PBC	8 (2.7)	5 (4.8)	3 (1.6)
Alcohol	12 (4.1)	11 (10.6)	1 (0.5)
Others	93 (31.8)	52 (50.0)	41 (21.8)
Child-Pugh			
A	245 (83.9)	84 (80.8)	161 (85.6)
B	47 (16.1)	20 (19.2)	27 (14.4)
Platelets (10^3^/*μ*L)	111 (80-162.5)	97 (68-139.5)	118.5 (88.5-171)
ALT (IU/L)	26.9 (19-41.9)	22 (16-29.5)	31.5 (21.3-50)
ALB (g/L)	41 (36.3-44.1)	37.5 (34.5-41.1)	42.2 (38.5-45.7)
Bilirubin (*μ*M/L)	20.1 (14-30.1)	21 (15.1-30.6)	19 (14-28.8)
INR	1.2 (1.1-1.3)	1.2 (1.1-1.4)	1.2 (1.1-1.3)
TE^**†**^ LSM (kPa)	19.8 (12-34.8)	19.8 (12-34.8)	NA
High-risk varices (%)	47 (16.1)	19 (18.3)	28 (14.9)
Any varices	142 (48.6)	54 (51.9)	88 (46.8)
With RESIST-HCV criteria	109	26	83
With RESIST-HCV criteria who had HRV	1	1	0

Continuous variables are expressed as median (interquartile range) unless indicated. ^†^TE was available in 90 patients. Abbreviations: ALB: albumin; ALT: alanine aminotransferase; HRV: high-risk varices; INR: international normalized ratio; LSM: liver stiffness measurement; PBC: primary biliary cholangitis; TE: transient elastography.

**Table 2:** Comparison of the total population with HRV vs. the population without HRV.

Variables	HRV, *N* = 47	Non-HRV, *n* = 245	*P* ^∗^
Male (%)	30 (63.8)	157 (64.1)	0.974^a^
Age (years)	50 (44.5-58)	53 (43-61)	0.697
BMI	22 (21.1-23.4)	23.2 (21.5-25.2)	0.126
Etiology			0.363^a^
Hepatitis B	34	140	
Hepatitis C	0	4	
PBC	1	7	
Alcohol	2	10	
Others	10	83	
Child-Pugh			0.291^a^
A	37	208	
B	10	37	
Platelets (10^3^/*μ*L)	77 (57.5-96)	119 (90-169)	<0.001
ALT (IU/L)	29.3 (21-37)	26.1 (18-42)	0.465
ALB (g/L)	37.2 (33.2-40.9)	41.6 (36.8-44.4)	<0.001
Bilirubin (*μ*M/L)	22.3 (15.5-33.2)	20 (14-28.1)	0.129
INR	1.3 (1.2-1.4)	1.2 (1.1-1.3)	<0.001
TE^**†**^ LSM (kPa)	30.1 (14-35.6)	19.6 (12-34.8)	0.405

^∗^Statistical comparison between the presence and absence of high-risk varices using Mann–Whitney *U* test unless indicated. ^a^Statistical comparison between the presence and absence of high-risk varices using Chi-square test or Fisher's exact test. ^**†**^TE was available in 90 patients. Abbreviations: ALB: albumin; HCV: hepatitis C virus; INR: international normalized ratio; LSM: liver stiffness measurement; PBC: primary biliary cholangitis; TE: transient elastography.

**Table 3:** Need for EGD based on noninvasive criteria for ruling out high-risk varices.

	Pooled cohort (292patients)		
Characteristics	B6C (*n* = 90)	EB6C (*n* = 90)	RESIST-HCV (*n* = 292)
AUROC	0.599	0.615	0.710
^a^Spared EGD, *n* (%)	14 (15.6)	21 (23.3)	109 (37.3)
^b^Missed HRV, *n* (%)	0 (0)	1 (4.8)	1 (0.9)
NPV (%)	100	95.2	99.1
Sen (%)	100	94.7	97.9
Spe (%)	19.7	28.2	44.1
PPV (%)	25	26.1	25.1

Abbreviations: AUROC: area under receiving operator characteristics curve; B6C: Baveno VI criteria; EB6C: expanded Baveno VI criteria; EGD: esophagogastroduodenoscopy; HRV: high-risk varices; NPV: negative predictive value; PPV: positive predictive value; Sen: sensitivity; Spe: specificity. ^a^The spared EGD rate was calculated as the ratio between the number of patients with EGD that could be spared and the total number of patients. ^b^The missed HRV rate was defined as the rate of patients with missed HRV among the patients with spared EGD.

## Data Availability

The original data can be obtained from the correspondence author.

## Abbreviations

ALT: Alanine aminotransferase
LSM: Liver stiffness measurement
TE: Transient elastography
HRV: High-risk varices
HCV: Hepatitis C virus
EGD: Esophagogastroduodenoscopy
PPV: Positive predictive value
NPV: Negative predictive value
pH: Portal hypertension
GEV: Gastroesophageal varices
NITs: Noninvasive tests.
